# In Situ-Initiated Poly-1,3-dioxolane Gel Electrolyte for High-Voltage Lithium Metal Batteries

**DOI:** 10.3390/molecules29112454

**Published:** 2024-05-23

**Authors:** Mingyang Xin, Yimu Zhang, Zhenhua Liu, Yuqing Zhang, Yutong Zhai, Haiming Xie, Yulong Liu

**Affiliations:** School of Chemistry, Northeast Normal University, Changchun 130024, China; xinmy608@nenu.edu.cn (M.X.); zhangym2819@163.com (Y.Z.); liuzh465@nenu.edu.cn (Z.L.); zhangyq902@nenu.edu.cn (Y.Z.); zhaiyutonga@163.com (Y.Z.)

**Keywords:** lithium metal battery, gel polymer electrolyte, low temperatures, high-voltage cathode

## Abstract

To realize high-energy-density Li metal batteries at low temperatures, a new electrolyte is needed to solve the high-voltage compatibility and fast lithium-ion de-solvation process. A gel polymer electrolyte with a small-molecular-weight polymer is widely investigated by combining the merits of a solid polymer electrolyte (SPE) and liquid electrolyte (LE). Herein, we present a new gel polymer electrolyte (P-DOL) by the lithium difluoro(oxalate)borate (LiDFOB)-initiated polymerization process using 1,3-dioxolane (DOL) as a monomer solvent. The P-DOL presents excellent ionic conductivity (1.12 × 10^−4^ S cm^−1^) at −20 °C, with an oxidation potential of 4.8 V. The Li‖LiCoO_2_ cell stably cycled at 4.3 V under room temperature, with a discharge capacity of 130 mAh g^−1^ at 0.5 C and a capacity retention rate of 86.4% after 50 cycles. Moreover, a high-Ni-content LiNi_0.8_Co_0.1_Mn_0.1_O_2_ (NCM811) cell can steadily run for 120 cycles at −20 °C, with a capacity retention of 88.4%. The underlying mechanism of high-voltage compatibility originates from the dense and robust B- and F-rich cathode interface layer (CEI) formed at the cathode interface. Our report will shed light on the real application of Li metal batteries under all-climate conditions in the future.

## 1. Introduction

The utilization of electric vehicles in high-latitude regions necessitates lithium metal batteries that can operate stably in a low-temperature range. Also, to alleviate the drive-range anxiety, Li metal batteries coupled with high-voltage cathodes have become urgent [1,2,3,4]. Unfortunately, commercial electrolytes, which feature an ethylene carbonate (EC) component with a melting point of 36.4 °C, are prone to crystallize at colder temperatures [5]. This crystallization results in a drastic decrease in terms of ionic conductivity, even blocking the porous structure of the electrode, thus compromising their overall performance [6,7,8]. Therefore, low-freezing-point solvents such as 1,3-dioxolane (DOL)-based electrolytes for ultra-low-temperature lithium metal batteries have been proposed in recent years. Nevertheless, DOL-based electrolytes still face some challenges before practical applications. Due to the narrow electrochemical window of DOL solvent, α-H is easily oxidized at high voltage by nucleophiles such as single oxygen or superoxide species, making it unable to couple with a layered-structure cathode [9,10]. Except for the cathode interface, the unstable solid electrolyte interface (SEI) formed on the surface of Li metal in a liquid-based DOL electrolyte leads to the formation of Li dendrites at high current densities [11]. Moreover, the low boiling point and high flammability of DOL also pose significant safety risks for batteries, hindering the scale-up process. Solid-state electrolytes exhibit significant advantages due to their high safety, wide operating temperature range, and excellent electrochemical stability [12]. A safe polymer electrolyte represents a promising candidate solution for high-voltage and safe Li metal batteries. Traditional solid polymer electrolytes (SPEs) are efficient in suppressing lithium dendrite growth, thereby improving battery safety [13], but they exhibit poor interfacial compatibility and lower room-temperature lithium-ion conductivity due to their ion-conduction mechanism, which does not meet the practical demands of batteries [14]. In contrast, gel polymer electrolytes (GPEs) arise as an ideal choice for developing high-performance lithium metal batteries due to their high ionic conductivity and good interfacial contact with electrodes [15,16]. However, the practical application process is hindered by the persistent challenges posed by its inadequate mechanical strength and considerable thickness [17]. Recent studies have shown that a simple in situ polymerization route can be directly applied to the battery industry, balancing the ionic conductivity and interfacial issues [18]. On the one hand, the precursor solution effectively infiltrates the cathode and anode, achieving lower interfacial resistance. On the other hand, the gel polymer electrolyte (GPE) enhances the safety of the battery [19,20,21,22,23]. Nevertheless, most of the in situ polymerization processes reported in the literature require additional initiators to prepare GPEs, which may introduce some intrinsic defects [24,25,26,27,28]. For instance, 2,2′-Azobis (2-methylpropionitrile) (AIBN), a common initiator for free radical polymerization, may potentially undergo side reactions, thus adversely impacting battery performance [29]. Meanwhile, the application of GPEs in a low-temperature environment may also slow down the reaction speed between solid-state electrolytes and electrodes, which affects the rate performance of batteries, thereby deteriorating their cycling performance [4,30,31,32,33].

Herein, we attempted the in situ polymerization of DOL within a polypropylene (PE) separator. Lithium bis(fluorosulfonyl)imide (LiFSI) was chosen as the primary salt because it usually decomposes to form LiF at the anode side, thus ensuring the stability of the lithium metal interface. Meanwhile, lithium difluoro(oxalate)borate (LiDFOB) was selected as a bifunctional additive. In one aspect, LiDFOB as a lithium salt can regulate the electrode/electrolyte interface, which usually preferentially forms a B-rich CEI on the cathode side to improve the high-voltage stability of the electrolyte [34]. From a second aspect, it can directly initiate the ring-opening polymerization reaction, which avoids the introduction of impurities. In addition, the high solubility of LiDFOB gives it great potential to work at low temperatures. With these advantages, we mixed 0.6 mol L^−1^ (M) of LiFSI and 0.4 M LiDFOB in DOL solvent and prepared a poly-DOL gel electrolyte (P-DOL) by the in situ ring-opening reaction. The electrochemical tests demonstrated its excellent ionic conductivity (3.62 × 10^−4^ S cm^−1^), high lithium-ion transference number (0.61), and good compatibility with lithium metal. As a result, the Li‖LiFePO_4_ (LFP) cell presents a remarkably long cycling stability with a capacity retention of 82% at 1 C for 500 cycles. In addition, the oxidation resistance potential of P-DOL was improved to 4.8 V relative to the DOL-based electrolyte. The Li‖LiCoO_2_ (LCO) cells exhibited stable cycling performance at room temperature. It is worth highlighting that the P-DOL-based Li‖LiNi_0.8_Co_0.8_Mn_0.1_O_2_ (NCM811) cell retains a remarkable 74.5% of its room-temperature capacity even at −20 °C. This work offers broad prospects for the application of high-voltage, high-energy-density lithium metal batteries.

## 2. Results

### 2.1. Characterizations and Electrochemical Properties of P-DOL

**Figure 1:**
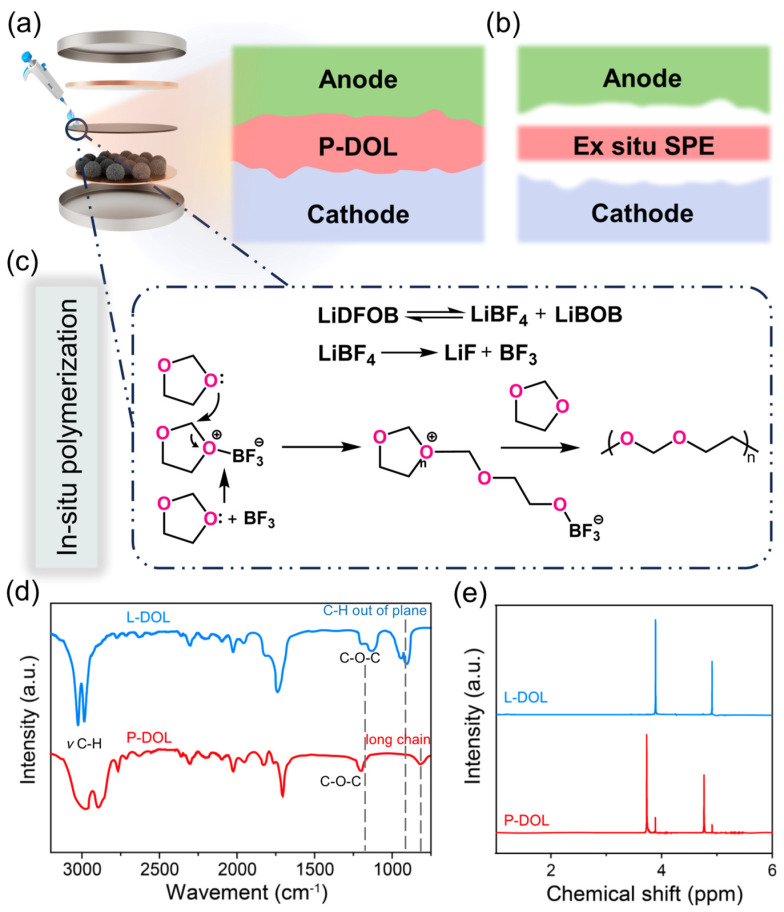
Schematic of (**a**) the in-situ polymerization, (**b**) the ex situ polymerization electrolyte processes, and (**c**) the reaction mechanism of in situ polymerization; (**d**) FTIR spectra of P-DOL and L-DOL from 800 to 3000 cm^−1^; (**e**) ^1^H NMR spectra of P-DOL and L-DOL.

Figure 1a depicts the process of the in situ polymerization method, where 30 μL liquid precursors were infiltrated into the cells, enabling a uniform polymerization of the electrolyte onto the electrodes. This leads to a tight electrode/electrolyte interfacial contact, simulating liquid electrolytes, which thoroughly wet the electrode surface and establish a connecting pathway for efficient ion transportation. In contrast, solid-state polymer electrolyte batteries assembled ex situ are usually plagued by high interfacial impedance (Figure 1b). For a deeper understanding of the polymerization process, Figure 1c shows the polymerization reaction mechanism in detail. LiDFOB undergoes a decomposition reaction, yielding BF_3_ [35], which serves as a Lewis acid initiator that attacks the oxygen atom of the monomer, resulting in the formation of the oxonium ion. Subsequently, repeated attacks of additional monomers on atoms adjacent to the carbon and oxonium ions trigger the formation of the copolymer chain, ultimately leading to the production of P-DOL.

To validate the synthesis of gel electrolytes, the functional groups of L-DOL and P-DOL initiated by LiDFOB were characterized by an FTIR test. As shown in Figure 1d, in the spectrum of L-DOL, the C-O-C vibration peak (1030 cm^−1^ to 1000 cm^−1^) and C-H vibration (954 cm^−1^, out of plane) were the characteristic peaks of solvent DOL. Compared to the L-DOL, the C-H vibration (954 cm^−1^, out of plane) was absent and a new peak at 845 cm^−1^ appeared corresponding to the group of -(CH_2_)- in the spectrum of P-DOL, which suggested the conversion of DOL from a cyclic monomer to a long-chain polymer, thereby confirming the occurrence of ring-opening polymerization. Subsequently, a Nuclear Magnetic Resonance (NMR) test was used to further investigate the chemical compositions of L-DOL and P-DOL, and the ^1^H NMR spectra are displayed in Figure 1e. L-DOL showed two main peaks at 3.87 and 4.88 ppm, which is consistent with previous reports of DOL monomers [29]. Specifically, two new peaks emerge in the spectrum of the P-DOL electrolyte. The lower peak at 3.71 ppm belongs to the functional group -O-CH_2_-CH_2_-O- and the higher chemical displacement at 4.68 ppm belongs to the functional group -O-CH_2_-O- [36]. The above results are consistent with previous FTIR spectral analysis. Therefore, based on the optical images and characterization results, it is proved that the LiDFOB can effectively induce DOL ring-opening polymerization.

**Figure 2:**
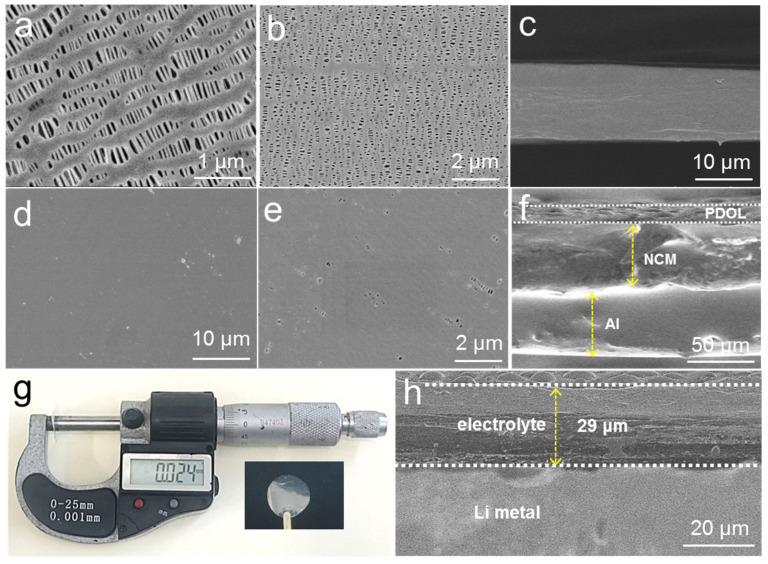
(**a**) Top-view, (**b**) bottom-view, and (**c**) cross-sectional SEM images of PE separator; (**d**,**e**) SEM images of P-DOL; (**f**) cross-sectional SEM image of NCM/P-DOL; (**g**) optical and thickness measurement photographs of P-DOL; (**h**) cross-sectional SEM image of Li/P-DOL.

The surface morphology and interface characteristics of electrolytes are important for the lithium-ion diffusion and charge transfer process. As illustrated in Figure 2a,b, the top- and bottom-view surface morphology images of the PE were full of uniform pores. The cross-sectional image of the PE shows that its thickness was about 12 μm (Figure 2c). The scanning electron microscopy (SEM) images of P-DOL formed through in situ polymerization (Figure 2d,e) reveal that its surface morphology was uniform and dense. Under higher magnification, only the smaller pores are visible, which indicates that the gel electrolyte evenly covered the separator. Figure 2f shows the cross-sectional SEM image of the electrolyte and cathode after in situ assembly of the batteries, where a close contact can be observed between the P-DOL and the NCM811, which enhanced the ionic conduction at the interface and avoided the loss of connectivity between the cathode particles and the electrolyte. Furthermore, the cross-sectional SEM images of P-DOL and Li metal revealed that the two layers maintained close contact, and the thickness of the electrolyte film was determined to be approximately 29 μm, which is consistent with the measurements obtained using a screw micrometer (Figure 2g,h). 

The importance of safety for electrolytes cannot be overstated; hence, fire tests were carried out to investigate the flammability of P-DOL and L-DOL. As shown in Figure S1, the L-DOL electrolyte ignited immediately with a self-extinguishing time (SET) reaching up to 92.0 s/g. In contrast, the SET value of the P-DOL was 8.2 s/g, suggesting that P-DOL demonstrates superior safety performance. The thermal stability of electrolytes was evaluated through the thermogravimetric test. We observed that the L-DOL electrolyte began to evaporate at room temperature, with full evaporation at nearly 40 °C. On the contrary, P-DOL only had a significant mass loss above 140 °C, indicating that the P-DOL exhibits better thermal stability compared to the liquid L-DOL (Figure S2).

### 2.2. Electrochemical Performances and Li^+^ Plating/Stripping Behavior

**Figure 3:**
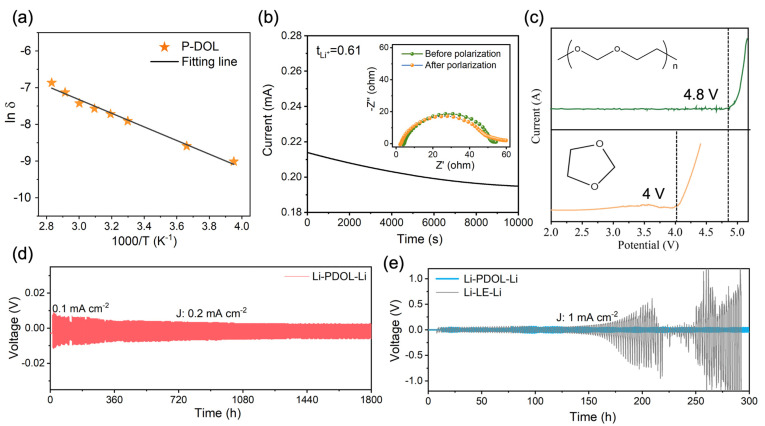
(**a**) Arrhenius plot of P-DOL ionic conductivity; (**b**) current–time curves and impedance plots of Li‖P-DOL‖Li cells before and after testing; (**c**) LSV curves of the P-DOL and L-DOL; (**d**) the galvanostatic cycling curve of the Li‖P-DOL‖Li cell at a constant current density of 0.2 mA cm^−2^; (**e**) polarization voltage profiles of P-DOL and LE symmetric cells at 1 mA cm^−2^.

The impedance of P-DOL at different temperatures was obtained through the electrochemical impedance spectroscopy (EIS) test and the ionic conductivity was calculated using Supplementary Information Equation (S1). The ionic conductivity of P-DOL at room temperature was 3.62 × 10^−4^ S cm^−1^, which is comparable to existing gel polymer electrolytes [37,38,39]. This indicates that P-DOL possesses significant potential as an electrolyte. And with the increase in temperature, the ionic conductivity under 40 °C, 50 °C, 60 °C, 70 °C, and 80 °C is 4.45 × 10^−4^, 5.17 × 10^−4^, 5.95 × 10^−4^, 8.01 × 10^−4^, and 1.16 × 10^−3^ S cm^−1^, respectively (Figure 3a). It is worth noting that the ionic conductivity of P-DOL at −20 °C could reach 1.12 × 10^−4^ S cm^−1^, enabling the batteries operating at low temperatures. The activation energy calculated by the Arrhenius equation (Supplementary Information Equation (S2)) was 0.16 eV. The relatively low activation energy indicated that the energy barrier for Li^+^ migration is small, enabling rapid lithium-ion migration [40].

The lithium transference number (*t*_Li_^+^) is another important parameter to evaluate the performance of an electrolyte, which responds to the percentage of ionic conductivity contributed by the movement of Li^+^. The constant-potential polarization method was employed to test the *t*_Li+_ of P-DOL. As depicted in Figure 3b, the *t*_Li+_ of P-DOL is 0.61, according to Equation (1). This high *t*_Li+_ is attributed to the anion being bound by the copolymer grid, preventing its migration over long distances [41,42]. The Linear Sweep Voltammetry (LSV) test was used to assess the electrochemical stability window of the electrolyte. As shown in Figure 3c, the oxidative decomposition of L-DOL occurred below 4.0 V (vs. Li/Li^+^). However, the oxidation potential of P-DOL increased to 4.8 V, a significant enhancement compared to L-DOL, which could be attributed to the transition of DOL from monomers to long chains and the addition of LiDFOB [29,34]. Therefore, P-DOL has the potential to be compatible with high-voltage cathode materials.

To evaluate the interface stability between the P-DOL and lithium metal, Li‖Li symmetric cells were assembled and the plating/stripping behaviors were studied. A critical current density (CCD) test was conducted to determine the maximum endurable current density of P-DOL, and the current density increased with a fixed step length (0.1 mA cm^−2^). As the current density increased, the polarization voltage also rose, leading to a short-circuit fault at a current density of 3.1 mA cm^−2^ (Figure S3). Subsequently, the cyclic stability of the Li‖P-DOL‖Li cell was investigated with a current density of 0.2 mA cm^−2^, as shown in Figure 3d. The cell could stabilize for more than 1800 h, accompanied by a low overpotential of 5.8 mV and a stable voltage platform (Figure S4a), suggesting the good lithium metal stability of P-DOL. Moreover, a comparative analysis of the Li‖Li cell was conducted on P-DOL and LE. At a current density of 1 mA cm^−2^, the Li‖LE‖Li cell exhibits an overpotential of 52 mV. As the cycling continues, the polarization voltage gradually rises after 150 h (Figure S4b), ultimately leading to a short-circuit at 210 h. In stark contrast, the Li‖P-DOL‖Li cell demonstrates remarkable stability for at least 300 h, thus emphasizing the superior stability of P-DOL with Li. Furthermore, a Nyquist impedance spectrum was obtained to evaluate the interface stability between P-DOL and lithium-metal-negative electrodes with storage time. The semicircles in the high-frequency and the intermediate-frequency regions in the spectrum represent the resistance of Li^+^ transport through the interfacial layer and charge transfer, respectively, where the interfacial resistance (R_i_) is mainly contributed by the SEI [43], and Figure S5 shows the equivalent circuit diagram of the EIS. As shown in Figure S6a, the initial R_i_ of the Li‖LE‖Li cell was 61.1 Ω. However, with the increase in shelving time, the R_i_ of the cell was 91.4 Ω, 113.8 Ω, 144.5 Ω, and 212.3 on the 1st, 3rd, 5th, and 7th days, respectively (Table S1). The R_i_ of the freshly assembled Li‖P-DOL‖Li cell was very low (33.4 Ω). As the storage time extends, the R_i_ increases slightly due to polymerization reactions and the formation of SEI, and then stabilizes at 53.6 Ω (Figure S6b). The above electrochemical test results indicated that P-DOL exhibits good compatibility with the Li metal anode, which enables close contact and good lithium-ion transport between the electrolyte and Li metal [44].

**Figure 4:**
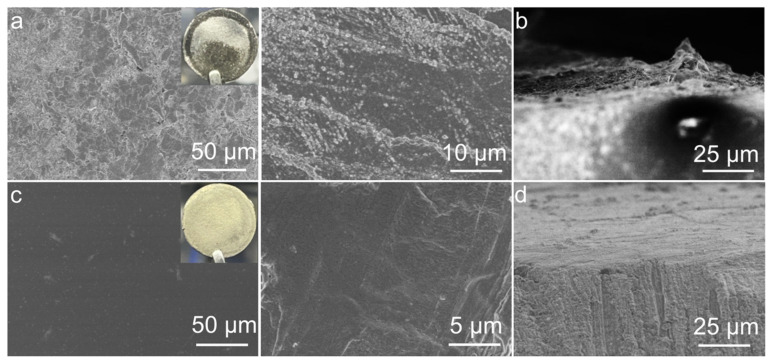
Optical photographs and SEM images of Li anode in (**a**,**c**) Li‖LE‖Li and (**b**,**d**) Li‖P-DOL‖Li cells after cycling.

After cycling, the Li‖LE‖Li cell was disassembled, and a significant amount of pulverized and detached lithium was observed (Figure 4a), showing a rough and loose appearance. Dendritic lithium growth can also be clearly seen from the cross-sectional SEM image (Figure 4b), which contributed to the unstable cycling performance from the LE-based symmetric cell. On the contrary, notable differences were observed in the Li-negative electrode after the Li‖P-DOL‖Li cell cycling. Both the optical and SEM images (Figure 4c) exhibited a very smooth and dense lithium surface morphology. The cross-sectional SEM images also revealed the absence of “dead lithium” (Figure 4d), indicating that P-DOL effectively suppresses lithium dendrite growth and thus achieves uniform plating/stripping behavior [45]. 

### 2.3. Full Cell Performance of P-DOL

**Figure 5:**
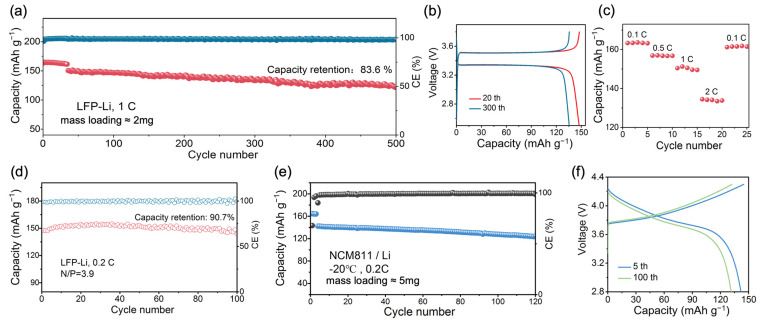
(**a**) Cycling performance of Li‖LFP at 1 C; (**b**) charge/discharge curve and (**c**) rate performance of Li‖LFP cell. (**d**) Capacity–efficiency plot of Li‖LFP cell with N/P = 3.9 at 0.2 C; (**e**) electrochemical performance of P-DOL at −20 °C; (**f**) charge/discharge curve of Li‖NCM811.

Galvanostatic charge/discharge measurements were used to evaluate the performance of the full Li‖P-DOL‖LFP cell. As displayed in Figure 5a, the cell presents a discharge capacity of 164.6 mAh g^−1^ and an average coulombic efficiency (CE) of 96.4% at 0.1 C. The discharge capacity reaches 150 mAh g^−1^ when the rate rises to 1 C. After 300 cycles, it can still achieve a capacity of 135 mAh g^−1^, with a capacity retention of 90%. Even after 500 cycles, the capacity retention rate remains at 83.6%, showing the good cycling stability. As indicated in Figure 5b, a typical and stable charge and discharge platform was maintained throughout the cycling process, along with an exceptionally low polarization voltage of 0.16 V. Additionally, the rate performance of the Li‖P-DOL‖LFP cell was tested, as shown in Figure 5c. The discharge specific capacities of the cell at 0.1, 0.5, 1, and 2 C were 163.3, 156.9, 150.3, and 134.3 mAh g^−1^, respectively. When it returned to 0.1 C, the reversible capacity returned to 161.2 mAh g^−1^, which was 98.7% of the capacity under the current of 0.1 C, indicating that the Li‖P-DOL‖LFP possessed an excellent rate performance.

Moreover, in pursuit of the practical requirements (limited lithium source and high-loading cathode), a full cell was assembled using LFP with a loading of 12 mg cm^−2^ as the cathode, a 50 μm thin lithium metal as the anode (N/P = 3.9), and 30 mL of P-DOL as the electrolyte. Subsequently, galvanostatic charge–discharge tests were conducted under real application conditions. As shown in Figure 5d, the cell presented an initial discharge capacity of 151 mAh g^−1^, a CE of 98.73% at 0.2 C, and a capacity retention rate of 90.7% after 100 cycles. The above excellent cycling and rate performances are attributed to the high ionic conductivity, *t*_Li+_, and stable lithium metal compatibility with P-DOL [46].

To investigate the compatibility between P-DOL and the layered structure cathode under high cut-off voltage, the Li‖LiCoO_2_ cells based on P-DOL and L-DOL were tested, respectively. As indicated in Figure S7a, the Li‖P-DOL‖LCO cell shows a high initial discharge ratio capacity of 158.6 mAh g^−1^ at 0.1 C, reaching 148 mAh g^−1^ at 0.2 C and 130 mAh g^−1^ at 0.5 C, and the capacity retention rate is 86.4% after 50 cycles. In contrast, the Li‖L-DOL‖LCO cell demonstrated an opposite result with complete failure within 15 cycles (Figure S7b), rendering it incompatible with high-voltage cathodes. This aligns closely with the previously mentioned LSV test results. The strategy of LiDFOB-induced DOL in situ polymerization can stabilize the cathode/electrolyte interface to a certain extent and improve the oxidation resistance of electrolytes [47], as further illustrated by our interface analysis.

**Figure 6:**
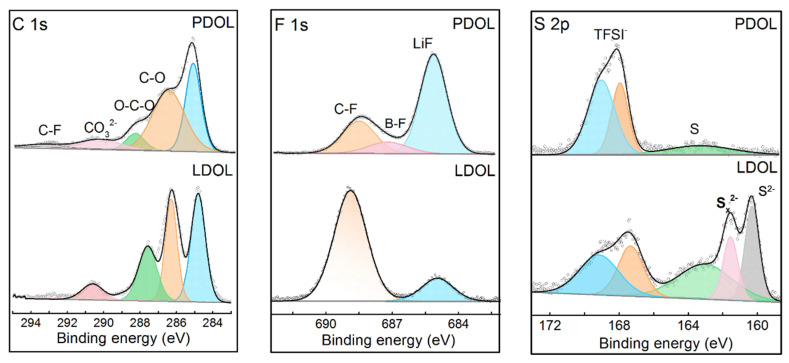
C 1s, F 1s, and S 2p XPS profiles of LCO from cycled Li‖P-DOL‖ LCO and Li‖L-DOL‖ LCO cells.

An X-ray photoelectron spectroscopy (XPS) test was performed on the LCO cathode of Li‖P-DOL‖LCO and Li‖L-DOL‖LCO cells after 50 cycles. Figure 6 illustrates the results of the XPS fine spectrum for the elements C 1s, F 1s, and S 1s. In the C 1s spectrum, organic components such as C-O (286.4 eV) and O-C-O (288.2 eV) can be observed, which were mainly formed by the decomposition of poly-DOL and DOL, where the content of organic components at the L-DOL/LCO interface was significantly higher than that of P-DOL. In addition, it can be seen that more LiF (684.8 eV) content was detected at the P-DOL/LCO interface from the F 1s spectrum, and LiF can effectively resist the continuous decomposition of the electrolyte [48]. More importantly, the peak of the B-F bond appears at 686.9 eV, a product of LiDFOB decomposition [49], which can effectively stabilize the cathode interface. In the S 2p spectrum, the L-DOL/LCO interface, in addition to the appearance of S-F bonds decomposed by TFSI, S^2−^, and S_x_^2−^ compounds, were also detected, which may increase the interfacial resistance and affect the cycling performance, whereas only a small amount of S species appeared at the P-DOL/LCO interface, due to a stable solvation structure [50]. From the above interface analysis, an organic–inorganic composite CEI at the P-DOL/LCO interface was formed, which hinders the occurrence of side reactions, thus improving the cycling performance of cells.

Electrolytes working at low temperatures have become urgently needed with the rapid growth of electrification in society. With an ionic conductivity of 1.12 × 10^−4^ S cm^−1^ at −20 °C, P-DOL has the potential to function effectively under low-temperature conditions. Therefore, the Li‖NCM811 cells were assembled and tested at −20 °C with the constant current charge/discharge. Surprisingly, as shown in Figure 5e, the Li‖P-DOL‖NCM811 cell achieved a discharge specific capacity of 141.6 mAh g^−1^ at −20 °C and 0.2 C (74.5% of the room-temperature capacity, much higher than traditional carbonate electrolyte of 40%). The capacity was maintained at 125.2 mAh g^−1^ after 120 cycles, with a capacity retention rate of 88.4% (Figure 5f), which outperforms the recently reported cycling performance of high-voltage batteries operating at low temperatures [39,51,52] (Table S2). More interestingly, the initial discharge specific capacity of the Li‖NCM811 reached 129 mAh g^−1^ at 0.5 C and −20 °C, and the capacity retention rate was 92.2% after 60 cycles (Figure S8). In addition, it is noteworthy that the electrolyte and lithium metal were stable at low temperatures, especially for the Li metal battery. Figure S9a shows the time–voltage curve of the Li‖P-DOL‖Li cell at −20 °C and 0.2 mA cm^−2^. Upon several cycles, the polarization voltage stabilizes at 19 mV for more than 1000 h, which indicated that P-DOL has an excellent stability against lithium metal under low-temperature conditions (Figure S9b). Thus, P-DOL can be used as the electrolyte in a wide range of temperatures with high stability.

**Figure 7:**
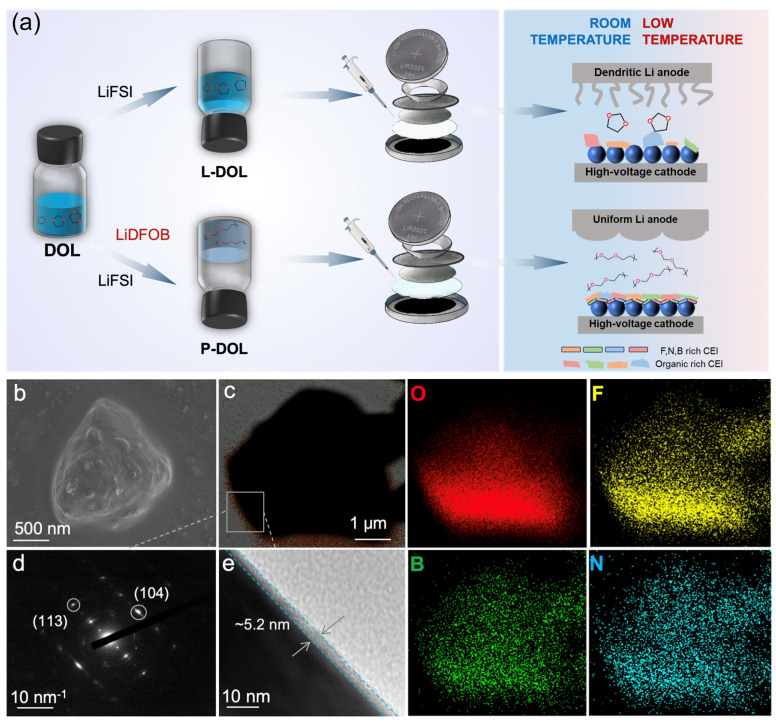
(**a**) Schematic diagram illustrating the synthesis of both L-DOL and P-DOL processes and their interfacial performance in successfully matching the high-voltage cathode with a broad temperature range. NCM811 electrode after cycling: (**b**) SEM image, (**c**) TEM-EDS elemental distribution, (**d**) SAED image, (**e**) high-resolution TEM image.

As depicted in Figure 7a, L-DOL exhibited irregular lithium dendrites on the anode along with an organic-rich CEI on the cathode. Conversely, P-DOL features uniform SEI formation on the anode and a CEI enriched with B, N, and F elements on the cathode. These distinct interfacial characteristics displayed notable differences in cell performance based on the two systems. To understand the interface stability at low temperature, SEM and transmission electron microscopy (TEM) tests were performed to observe the morphology/structure change of the NCM811 cathode particles undergoing cycling under low-temperature conditions. SEM images in Figure 7b revealed that the single-crystal particles of NCM811 have no cracks or surface damage, indicating the full protection of P-DOL on the top surface of the cathode. From the selected-area electron diffraction (SAED) image in Figure 7c, (104) and (113) diffraction spots of hcp crystals can be seen, which showed that the NCM811 crystals maintain the original structure (Figure 7d). In addition, from the high-resolution TEM image of Figure 7e, a uniform and dense CEI of about 5.2 nm can be observed. Such a good CEI can prevent the dissolution of Ni^2+^ in the cathode and limit the decomposition of electrolytes, thus ensuring good cycling stability. At the same time, the element distribution from energy-dispersion X-ray spectrometry–transmission electron microscopy (TEM-EDS) showed that the CEI with B and F was covered on the cathode surface, thereby protecting the cathode surface. 

## 3. Materials and Methods

### 3.1. Materials

1,3-Dioxalane (DOL) was purchased from Sigma-Aldrich (St. Louis, MO, USA). Lithium difluoro(oxalato)borate, and Lithium bis(fluorosulfonyl)imide were obtained from Aladdin (Shanghai, China).

### 3.2. Preparation of Electrolyte

Before configuring the electrolyte, the DOL solvent was placed into a sintered 4 Å molecular sieve for more than 36 h in order to remove the water and organic impurities; the LiFSI and LiDFOB were placed into a 120 °C vacuum oven for more than 12 h. An amount of 0.224 g of LiFSI was added to 2 mL of DOL solvent and stirred magnetically for 12 h at room temperature to obtain the liquid-DOL-based electrolyte (liquid-DOL-based electrolyte, abbreviated as L-DOL). Subsequently, 0.115 g of LiDFOB was added to the above electrolyte and stirred magnetically at a speed of 600 r min^−1^ for 1 h to obtain the precursor solution. After 6 h, P-DOL was obtained. As shown in Figure S10, the transformation of liquid precursors was promoted by LiDFOB into a uniform quasi-solid state within 6 h and remained stable after 24 h, while L-DOL without the initiator always maintained a liquid flow state, which means that we successfully synthesized P-DOL.

### 3.3. Electrochemical Measurements 

First, the P-DOL (40 μL) was infiltrated into the PE separator and the steel sheet was used as a blocking electrode. The EIS was performed at different temperatures. Then, the Li‖P-DOL‖SS and Li‖L-DOL‖SS cells were assembled and tested using LSV, with a scanning range of 0-6 V and a scanning speed of 0.1 mV s^−1^. The lithium metal symmetric cells (Li‖P-DOL‖Li) were assembled with P-DOL to perform the DC polarization test. The initial and steady-state currents are denoted as I_0_ and I_ss_, respectively. The initial and final interface resistances are denoted as R_0_ and R_s_, respectively. The *t_Li_^+^* was calculated using the Bruce–Vincent–Evans equation.

(1) $$t_{Li^{+}}=\frac{I_{SS}}{I_{0}}\left[\frac{∆V-I_{0}{R_{E}^{0}}_{before}}{∆V-I_{SS}{R_{E}^{SS}}_{after}}\right]$$

The Princeton (P4000) electrochemical workstation (AMETEK Co., Ltd., San Luis Obispo, CA, USA) was used to test lithium symmetric cells in the original state and lithium symmetrical cells for 1 day, 3 days, 5 days, and 7 days, respectively, with a frequency range of 1 M Hz~0.1 Hz.

The critical current density test of Li‖P-DOL‖Li cells was performed to increase the current at a rate of current density of 0.1 mA cm^−2^. The cycle stability of Li-symmetric cells was measured under the conditions of a current density of 0.2 mA cm^−2^ and a capacity of 0.2 mAh cm^−2^. The Li-symmetric cells were assembled with P-DOL and 1M LiPF_6_ EC/DEC (LE), and a current charge and discharge test was performed at a current density of 1 mA cm^−2^. The Li‖P-DOL‖LFP cells were assembled and a constant current charge and discharge test was performed from 2.5 to 3.8 V. The rate test of the full cells was performed at 0.1 C, 0.2 C, 0.5 C, 1 C, and 2 C. The full cells were assembled with 10 mg cm^−2^ of the LFP-positive electrode and 50 μm of the Li-metal-negative electrode and P-DOL, and they were tested at 2.5–3.8 V and 0.2 C. The Li‖P-DOL‖LCO and Li‖L-DOL‖LCO cells were assembled to compare the high-voltage stability of the two electrolytes, with a test voltage range of 2.8–4.3 V. The Li‖P-DOL‖NCM811 cells were assembled and tested at −20 °C and 0.2 C. All cells were assembled in a glove box filled with Ar and tested using a NEWARE battery tester.

### 3.4. Characterizations

The FTIR spectra of electrolytes in a wavelength range of 800–3000 cm^−1^ were obtained using a Nicolet-6700 infrared spectrometer. The prepared electrolytes were dissolved in dimethyl sulfoxide-d6 for ^1^H NMR analysis using a Bruker Avance III HD NMR spectrometer (Zurich, Switzerland). Thermogravimetric analysis was performed using an STA449F5 synchronous thermal analyzer under argon atmosphere. The thermal ramp consisted of 10 °C min^−1^, from room temperature to 450 °C. SEM (Hitachi S5500, Tokyo, Japan) was used to analyze the surface morphology of the prepared P-DOL electrolyte.

The Li‖Li cells assembled with P-DOL and LE were disassembled in the glove box and the surface shape of the recycled lithium metal was compared through an SEM test. The Li‖P-DOL‖LCO and Li‖L-DOL‖LCO cells were disassembled after cycling. The XPS test was performed on the LCO to characterize the chemical components of CEI. TEM (JEM-2100 PLUS) and SAED tests were performed on the NCM811 cathode after low-temperature cycling to observe the lattice structure of the cathode and the morphology of CEI. TEM-EDS was used to further determine the elemental distribution of the interface layer. XPS (Thermo ESCALAB250Xi, Waltham, MA, USA) was used to analyze the surface structure and chemistry of the post-cycled sodium metal electrodes. Before the above measurements, the surface of the electrodes was rinsed by 1,2-dimethoxyethane (DME) and then naturally dried to remove the residual solvents on the surface.

## 4. Conclusions

In summary, we have carefully designed a gel polymer electrolyte utilizing lithium salt (LiDFOB) to directly initiate the ring-opening polymerization of DOL. The commercially available in situ polymerization strategy not only enhances the electrochemical window of the electrolyte, but also ensures its interfacial compatibility with high-voltage cathodes and lithium metal anodes. As a result, the LiFePO_4_‖Li cell employing a cathode with a loading of 10 mg cm^−2^ and a 50 μm Li sheet as the anode (N/P = 3.9) maintains a capacity of 90.7% after 100 cycles at 0.2 C. The as-assembled 4.3 V LCO‖Li cell delivers a discharge capacity of 148 mAh g^−1^ which is sustained for 50 cycles at room temperature. This excellent performance is mainly ascribed to the excellent electrode/electrolyte interface (CEI/SEI) formed on LCO and Li simultaneously. Notably, the electrolyte exhibits remarkable performance even at low temperatures, evidenced by the NCM811‖Li cell achieving a capacity retention of 74.5% after 100 cycles at −20 °C. Our research can potentially be extended to other rechargeable wide-temperature solid-state batteries and provides a valuable framework for practical electrolyte engineering.

## Data Availability

Data are contained within the article and Supplementary Materials.

## Supplementary Materials

The following supporting information can be downloaded at https://www.mdpi.com/article/10.3390/molecules29112454/s1.

## References

[1] Lai P., Deng X., Zhang Y., Li J., Hua H., Huang B., Zhang P., Zhao J. (2023). Bifunctional Localized High-Concentration Electrolyte for the Fast Kinetics of Lithium Batteries at Low Temperatures. ACS Appl. Mater. Interfaces.

[2] Wang Q., Liu B., Shen Y., Wu J., Zhao Z., Zhong C., Hu W. (2021). Confronting the Challenges in Lithium Anodes for Lithium Metal Batteries. Adv. Sci..

[3] Wang Z., Sun Z., Shi Y., Qi F., Gao X., Yang H., Cheng H., Li F. (2021). Ion-Dipole Chemistry Drives Rapid Evolution of Li Ions Solvation Sheath in Low-Temperature Li Batteries. Adv. Energy Mater..

[4] Wu D., Chen L., Li H., Wu F. (2023). Solid-State Lithium Batteries-from Fundamental Research to Industrial Progress. Prog. Mater. Sci..

[5] Cheng L., Wang Y., Yang J., Tang M., Zhang C., Zhu Q., Wang S., Li Y., Hu P., Wang H. (2023). An Ultrafast and Stable Li-Metal Battery Cycled at −40 °C. Adv. Funct. Mater..

[6] Qin M., Zeng Z., Wu Q., Yan H., Liu M., Wu Y., Zhang H., Lei S., Cheng S., Xie J. (2023). Dipole–Dipole Interactions for Inhibiting Solvent Co-Intercalation into a Graphite Anode to Extend the Horizon of Electrolyte Design. Energy Environ. Sci..

[7] Su H., Liu P., Liu Y., Liu S., Zhong Y., Xia X., Wang X., Tu J. (2023). Fine-Tuning Li+ Solvation Structure Enabled by Steric Effect and Solvating Chemistry of the Anion for Practical Electrolyte Engineering. Nano Energy.

[8] Lu D., Zhang S., Li J., Huang L., Zhang X., Xie B., Zhuang X., Cui Z., Fan X., Xu G. (2023). Transformed Solvation Structure of Noncoordinating Flame-Retardant Assisted Propylene Carbonate Enabling High Voltage Li-Ion Batteries with High Safety and Long Cyclability. Adv. Energy Mater..

[9] Yi X., Li X., Zhong J., Wang Z., Guo H., Peng W., Duan J., Wang D., Wang J., Yan G. (2023). Uncovering the Redox Shuttle Degradation Mechanism of Ether Electrolytes in Sodium-Ion Batteries and Its Inhibition Strategy. Small.

[10] Li A.-M., Borodin O., Pollard T.P., Zhang W., Zhang N., Tan S., Chen F., Jayawardana C., Lucht B.L., Hu E. (2024). Methylation Enables the Use of Fluorine-Free Ether Electrolytes in High-Voltage Lithium Metal Batteries. Nat. Chem..

[11] Li Z., Yu R., Weng S., Zhang Q., Wang X., Guo X. (2023). Tailoring Polymer Electrolyte Ionic Conductivity for Production of Low- Temperature Operating Quasi-All-Solid-State Lithium Metal Batteries. Nat. Commun..

[12] Zhao Q., Stalin S., Zhao C.-Z., Archer L.A. (2020). Designing Solid-State Electrolytes for Safe, Energy-Dense Batteries. Nat. Rev. Mater..

[13] Zhang H., Li C., Piszcz M., Coya E., Rojo T., Rodriguez-Martinez L.M., Armand M., Zhou Z. (2017). Single Lithium-Ion Conducting Solid Polymer Electrolytes: Advances and Perspectives. Chem. Soc. Rev..

[14] Zhao Y., Wang L., Zhou Y., Liang Z., Tavajohi N., Li B., Li T. (2021). Solid Polymer Electrolytes with High Conductivity and Transference Number of Li Ions for Li-Based Rechargeable Batteries. Adv. Sci. Weinh. Baden-Wurtt. Ger..

[15] Ma Y., Sun Q., Wang S., Zhou Y., Song D., Zhang H., Shi X., Zhang L. (2022). Li Salt Initiated In-Situ Polymerized Solid Polymer Electrolyte: New Insights via in-Situ Electrochemical Impedance Spectroscopy. Chem. Eng. J..

[16] Jiang H., Yang C., Chen M., Liu X., Yin L., You Y., Lu J. (2023). Electrophilically Trapping Water for Preventing Polymerization of Cyclic Ether Towards Low-Temperature Li Metal Battery. Angew. Chem. Int. Ed..

[17] Tsao C.-H., Hsiao Y.-H., Hsu C.-H., Kuo P.-L. (2016). Stable Lithium Deposition Generated from Ceramic-Cross-Linked Gel Polymer Electrolytes for Lithium Anode. ACS Appl. Mater. Interfaces.

[18] Liu Q., Sun Y., Wang S., An Q., Duan L., Zhao G., Wang C., Doyle-Davis K., Guo H., Sun X. (2023). Highly Adaptable SEI/CEI Interfacial Layers Enabling Remarkable Performance of High-Nickel Solid-State Batteries. Mater. Today.

[19] Nie L., Chen S., Zhang M., Gao T., Zhang Y., Wei R., Zhang Y., Liu W. (2024). An In-Situ Polymerized Interphase Engineering for High-Voltage All-Solid-State Lithium-Metal Batteries. Nano Res..

[20] Vijayakumar V., Anothumakkool B., Kurungot S., Winter M., Nair J.R. (2021). In Situ Polymerization Process: An Essential Design Tool for Lithium Polymer Batteries. Energy Environ. Sci..

[21] Peng H., Long T., Peng J., Chen H., Ji L., Sun H., Huang L., Sun S. (2024). Molecular Design for In-Situ Polymerized Solid Polymer Electrolytes Enabling Stable Cycling of Lithium Metal Batteries. Adv. Energy Mater..

[22] Liu Q., Wang L., He X. (2023). Toward Practical Solid-State Polymer Lithium Batteries by In Situ Polymerization Process: A Review. Adv. Energy Mater..

[23] Sun M., Zeng Z., Peng L., Han Z., Yu C., Cheng S., Xie J. (2021). Ultrathin Polymer Electrolyte Film Prepared by in Situ Polymerization for Lithium Metal Batteries. Mater. Today Energy.

[24] Dong S., Sheng L., Wang L., Liang J., Zhang H., Chen Z., Xu H., He X. (2023). Challenges and Prospects of All-Solid-State Electrodes for Solid-State Lithium Batteries. Adv. Funct. Mater..

[25] Gao X., Xing Z., Wang M., Nie C., Shang Z., Bai Z., Dou S.X., Wang N. (2023). Comprehensive Insights into Solid-State Electrolytes and Electrode-Electrolyte Interfaces in All-Solid-State Sodium-Ion Batteries. Energy Storage Mater..

[26] Sang J., Tang B., Pan K., He Y.-B., Zhou Z. (2023). Current Status and Enhancement Strategies for All-Solid-State Lithium Batteries. Acc. Mater. Res..

[27] Xu L., Tang S., Cheng Y., Wang K., Liang J., Liu C., Cao Y.-C., Wei F., Mai L. (2018). Interfaces in Solid-State Lithium Batteries. Joule.

[28] Bonnick P., Muldoon J. (2022). The Quest for the Holy Grail of Solid-State Lithium Batteries. Energy Environ. Sci..

[29] Zhao Q., Liu X., Stalin S., Khan K., Archer L.A. (2019). Solid-State Polymer Electrolytes with in-Built Fast Interfacial Transport for Secondary Lithium Batteries. Nat. Energy.

[30] Randau S., Weber D.A., Kötz O., Koerver R., Braun P., Weber A., Ivers-Tiffée E., Adermann T., Kulisch J., Zeier W.G. (2020). Benchmarking the Performance of All-Solid-State Lithium Batteries. Nat. Energy.

[31] Zeng Z., Cheng J., Li Y., Zhang H., Li D., Liu H., Ji F., Sun Q., Ci L. (2023). Composite Cathode for All-Solid-State Lithium Batteries: Progress and Perspective. Mater. Today Phys..

[32] Li J., Ji Y., Song H., Chen S., Ding S., Zhang B., Yang L., Song Y., Pan F. (2022). Insights Into the Interfacial Degradation of High-Voltage All-Solid-State Lithium Batteries. Nano-Micro Lett..

[33] Manthiram A., Yu X., Wang S. (2017). Lithium Battery Chemistries Enabled by Solid-State Electrolytes. Nat. Rev. Mater..

[34] Mao M., Huang B., Li Q., Wang C., He Y.-B., Kang F. (2020). In-Situ Construction of Hierarchical Cathode Electrolyte Interphase for High Performance LiNi0.8Co0.1Mn0.1O2/Li Metal Battery. Nano Energy.

[35] Mateva R., Wegner G., Lieser G. (1973). Growth of Polyoxymethylene Crystals during Cationic Polymerization of Trioxane in Nitrobenzene. J. Polym. Sci. Polym. Lett. Ed..

[36] Liu F.-Q., Wang W.-P., Yin Y.-X., Zhang S.-F., Shi J.-L., Wang L., Zhang X.-D., Zheng Y., Zhou J.-J., Li L. (2018). Upgrading Traditional Liquid Electrolyte via in Situ Gelation for Future Lithium Metal Batteries. Sci. Adv..

[37] Wang Y., Chen S., Li Z., Peng C., Li Y., Feng W. (2022). In-Situ Generation of Fluorinated Polycarbonate Copolymer Solid Electrolytes for High-Voltage Li-Metal Batteries. Energy Storage Mater..

[38] Zheng J., Zhang W., Huang C., Shen Z., Wang X., Guo J., Li S., Mao S., Lu Y. (2022). In-Situ Polymerization with Dual-Function Electrolyte Additive toward Future Lithium Metal Batteries. Mater. Today Energy.

[39] Yuan B., Luo G., Liang J., Cheng F., Zhang W., Chen J. (2019). Self-Assembly Synthesis of Solid Polymer Electrolyte with Carbonate Terminated Poly(Ethylene Glycol) Matrix and Its Application for Solid State Lithium Battery. J. Energy Chem..

[40] Kondo Y., Abe T., Yamada Y. (2022). Kinetics of Interfacial Ion Transfer in Lithium-Ion Batteries: Mechanism Understanding and Improvement Strategies. ACS Appl. Mater. Interfaces.

[41] Shi Y., Hu L., Li Q., Sun Y., Duan Q., Jiang Y., Xu Y., Yi J., Zhao B., Zhang J. (2023). An Optimizing Hybrid Interface Architecture for Unleashing the Potential of Sulfide-Based All-Solid-State Battery. Energy Storage Mater..

[42] Yang G., Hou W., Zhai Y., Chen Z., Liu C., Ouyang C., Liang X., Paoprasert P., Hu N., Song S. (2023). Polymeric Concentrated Electrolyte Enables Simultaneous Stabilization of Electrode/Electrolyte Interphases for Quasi-solid-state Lithium Metal Batteries. EcoMat.

[43] Mc Carthy K., Gullapalli H., Ryan K.M., Kennedy T. (2021). Review—Use of Impedance Spectroscopy for the Estimation of Li-Ion Battery State of Charge, State of Health and Internal Temperature. J. Electrochem. Soc..

[44] Wang Z., Xie S., Gao X., Chen X., Cong L., Liu J., Xie H., Yu C., Liu Y. (2023). In-Situ Polymerized Carbonate Induced by Li-Ga Alloy as Novel Artificial Interphase on Li Metal Anode. Chin. Chem. Lett..

[45] Zhu X., Mo Y., Chen J., Liu G., Wang Y., Dong X. (2023). A Weakly-Solvated Ether-Based Electrolyte for Fast-Charging Graphite Anode. Chin. Chem. Lett..

[46] Hu T., Guo Y., Meng Y., Zhang Z., Yu J., Cai J., Yang Z. (2024). Uniform Lithium Deposition Induced by Copper Phthalocyanine Additive for Durable Lithium Anode in Lithium-Sulfur Batteries. Chin. Chem. Lett..

[47] Yang K., Li L., Xiao Y., Zhang Q., Xi C., Li B., Yu Y., Yang C. (2024). Congener-Derived Template to Construct Lithiophilic Organic-Inorganic Layer/Interphase for High Volumetric Capacity Dendrite-Free Li Metal Batteries. Chin. Chem. Lett..

[48] Zhang M., Liu R., Wang Z., Xing X., Liu Y., Deng B., Yang T. (2020). Electrolyte Additive Maintains High Performance for Dendrite-Free Lithium Metal Anode. Chin. Chem. Lett..

[49] Wu H., Tang B., Du X., Zhang J., Yu X., Wang Y., Ma J., Zhou Q., Zhao J., Dong S. (2020). LiDFOB Initiated In Situ Polymerization of Novel Eutectic Solution Enables Room-Temperature Solid Lithium Metal Batteries. Adv. Sci. Weinh. Baden-Wurtt. Ger..

[50] Park K., Yu S., Lee C., Lee H. (2015). Comparative Study on Lithium Borates as Corrosion Inhibitors of Aluminum Current Collector in Lithium Bis(Fluorosulfonyl)Imide Electrolytes. J. Power Sources.

[51] Yu J., Lin X., Liu J., Yu J.T.T., Robson M.J., Zhou G., Law H.M., Wang H., Tang B.Z., Ciucci F. (2022). In Situ Fabricated Quasi-Solid Polymer Electrolyte for High-Energy-Density Lithium Metal Battery Capable of Subzero Operation. Adv. Energy Mater..

[52] Zhao C., Zhao Q., Liu X., Zheng J., Stalin S., Zhang Q., Archer L.A. (2020). Rechargeable Lithium Metal Batteries with an In-Built Solid-State Polymer Electrolyte and a High Voltage/Loading Ni-Rich Layered Cathode. Adv. Mater..

