# Supplementary material for: In Situ-Initiated Poly-1,3-dioxolane Gel Electrolyte for High-Voltage Lithium Metal Batteries
Source: Molecules. 2024 May 23;29(11):2454. doi: 10.3390/molecules29112454 (PMC11173723; doi:10.3390/molecules29112454)
Supplement: Supplementary file 1 [file molecules-29-02454-s001.zip › molecules-3010314-supplementary.pdf]

High voltage cathode-based lithium-metal batteries for all-climate application with polymerized DOL electrolyte

Mingyang Xin[1,a], Yimu Zhang[1, a], Zhenhua Liu[1, a], Yuqing Zhang[1], Yutong Zhai[1], Haiming Xie[1*], Yulong Liu[1*]

[1]*School of Chemistry, Northeast Normal University, Changchun, 130024, China*
[a] *Co-first authors*
[*]*Corresponding author:* liuyl290@nenu.edu.cn; xiehm136@nenu.edu.cn

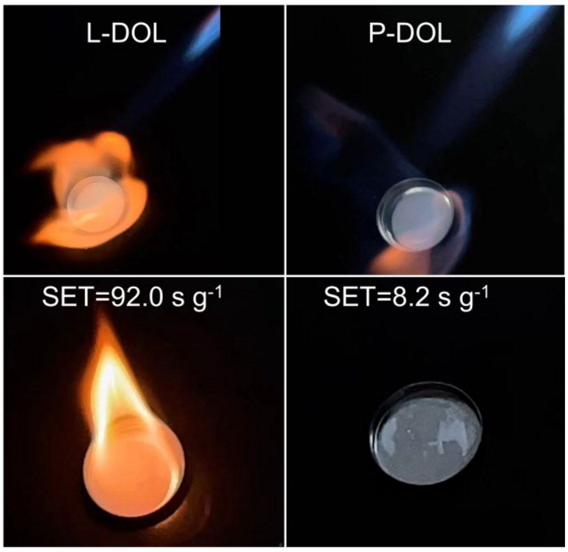

Figure S1. The Fire tests for P-DOL and L-DOL.

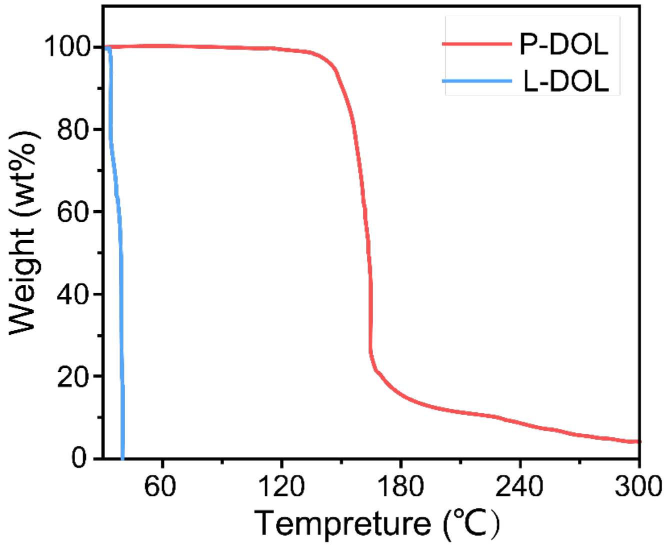

Figure. S2. Thermogravimetry curves of P-DOL and L-DOL

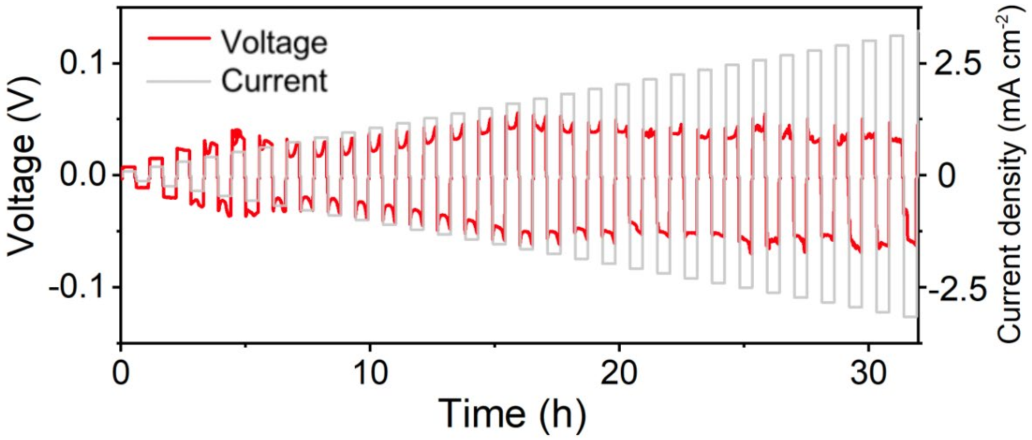

Figure. S3. Critical current density of Li‖PDOL‖Li cells.

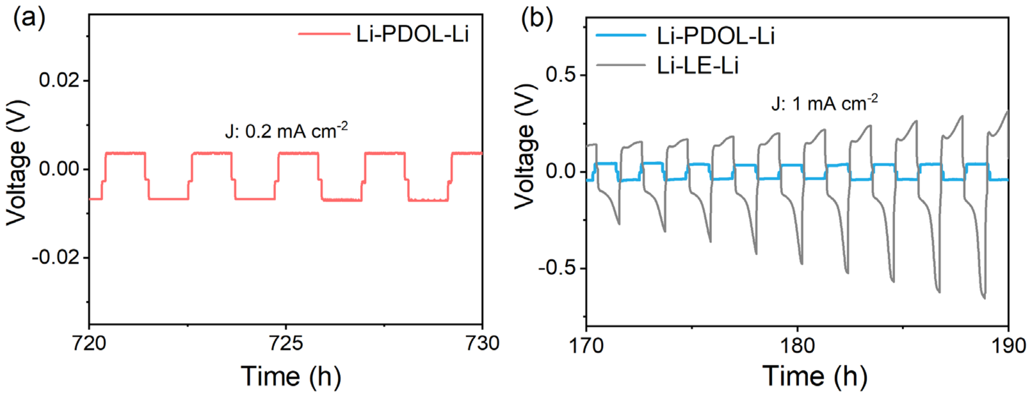

Figure. S4. The enlarged Charge-discharge curve of Li‖Li at 0.2 mA $cm^{-2}$and 1 mA cm^{-2}.

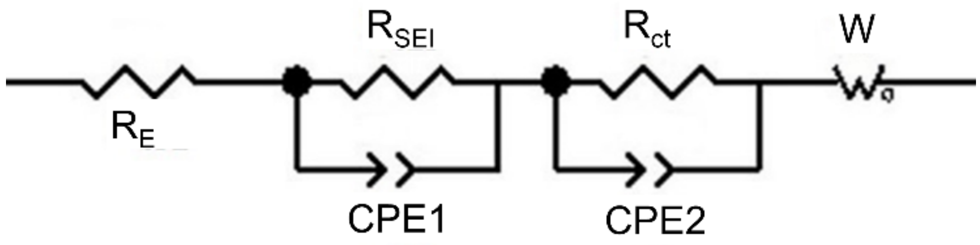

Figure. S5. The equivalent circuit of EIS

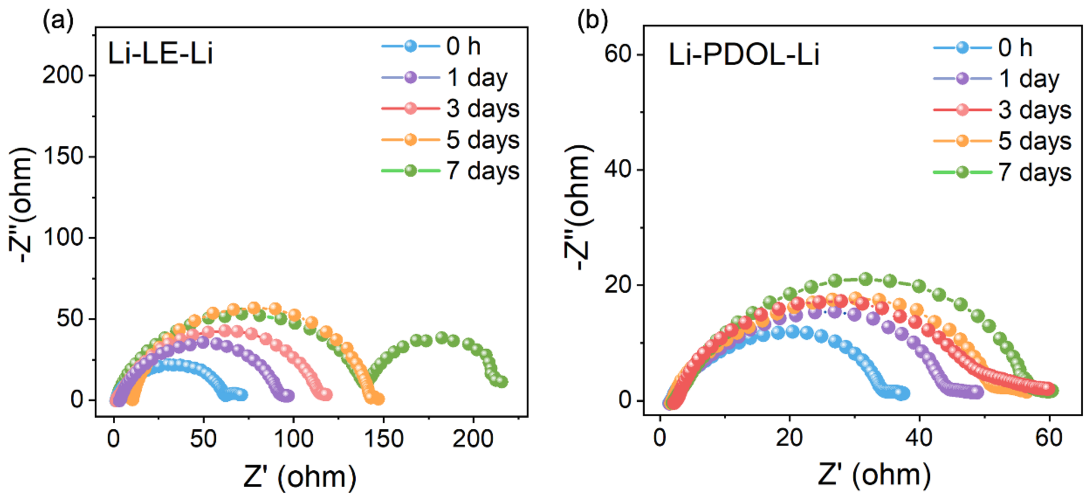

Figure. S6. Nyquist impedance plots of Li‖PDOL‖Li and Li‖LE‖Li batteries after 1 day, 3 days, 5 days and 7 days placement, respectively.

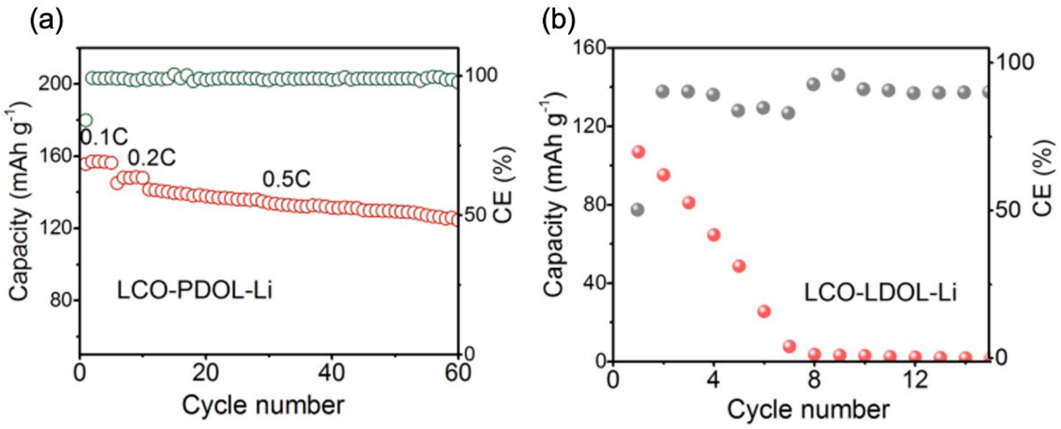

Figure. S7. Capacity-efficiency plots of (a) Li‖P-DOL‖LCO and (b) Li‖L-DOL‖LCO cells.

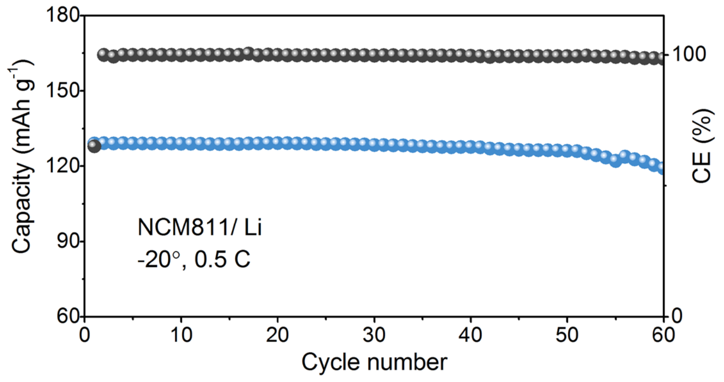

Figure S8. The Capacity-efficiency plot of the Li‖NCM811 cell at 0.5 C and -20 ºC.

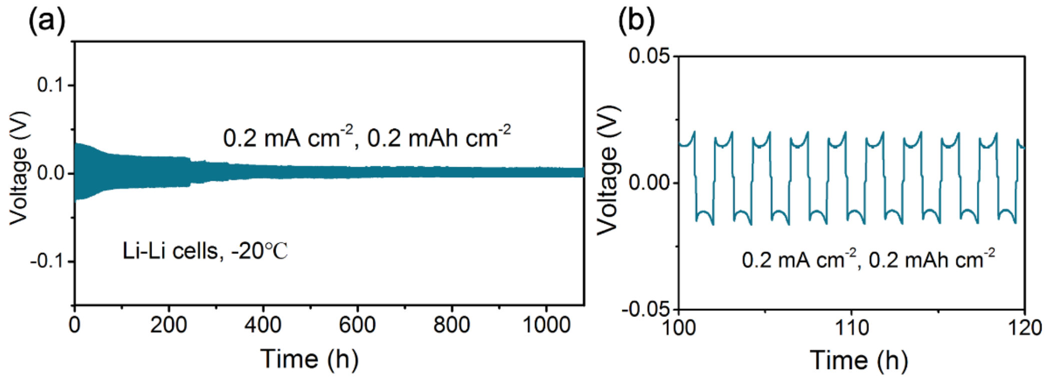

Figure. S9. (a) Charge-discharge curve of Li‖Li at 0.2 mA cm^{-2} and (b) zoomed-in figure

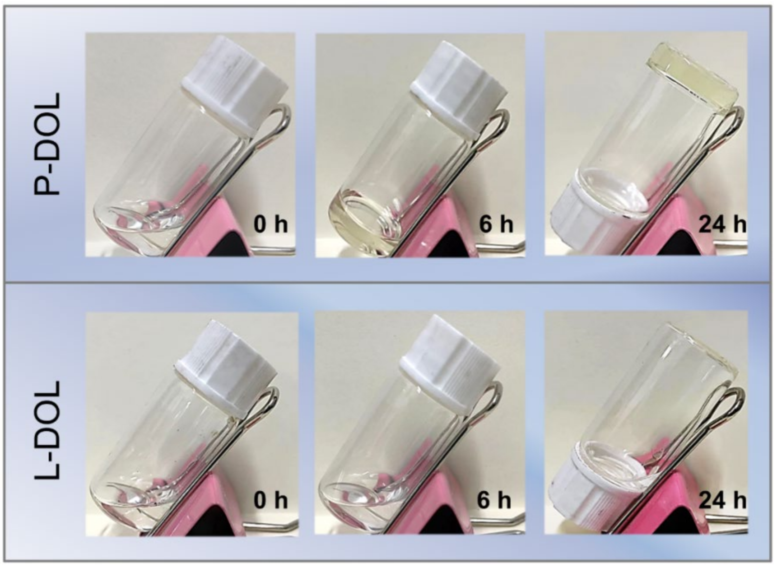

Figure. S10. Optical photographs of P-DOL and L-DOL at 0 h, standing for 6 h and 24 h

Table S1. Impedance values of Li‖Li batteries after 1, 3, 5 and 7 days of placement based on P-DOL and L-DOL.

R_i（Ω）	0 h	1 day	3 days	5 days	7 days
PDOL	33.4	42.6	48.3	51.1	53.6
LE	61.1	91.4	113.8	144.5	212.3

Table S2. Comparisons of the electrochemical performance in the previous literature.

**Cathode**	**electrolyte**	**Voltage range (V)**	**Capacity retention**	**Terperature**	**Ref**
NCM811	PDOL	2.8-4.3	0.2 C, 109 mAh g^{-1}, 100 cycles	-20 ºC	[51]
$LiFePO_4$	PEG-PISA	2.5-4.1	0.2 C, 62.5 mAh g^{-1}, 20 cycles	0ºC	[52]
NCM622	PDOL	2.8-4.3	0.1 C, 153 mAh g^{-1}, 60 cycles	25 ºC	[39]
NCM811	PDOL	2.8-4.3	0.2 C, 125.2 mAh g^{-1}, 120 cycles	-20 ºC	this work

Equations S1.

$$\delta = \frac{L}{R \times S} \qquad (S1)$$

where δ, L, R and S are the ionic conductivity, the thickness between the steel sheets, the electrochemical impedance and the contact area between the electrolyte and the blocking electrode, respectively.

Equations S2.

$$\sigma = AT^{-\frac{1}{2}} e^{-\frac{Ea}{R(T-T_0)}} \quad (S2)$$

where A， E_a， R and T_0 are the prefactor, apparent activation energy, ideal gas coefficient and ideal glass transition temperature， respectively.
